# A Case of Idiopathic Acute Pancreatitis in the First Trimester of Pregnancy

**DOI:** 10.1155/2015/469527

**Published:** 2015-12-30

**Authors:** Tomomi Hara, Haruhiko Kanasaki, Aki Oride, Tomoko Ishihara, Satoru Kyo

**Affiliations:** Department of Obstetrics and Gynecology, Shimane University School of Medicine, Izumo, Shimane 693-8501, Japan

## Abstract

Acute pancreatitis is rare in pregnancy, with an estimated incidence of approximately 1 in 1000 to 1 in 10,000 pregnancies. Acute pancreatitis in pregnancy usually occurs in the third trimester. Here, we report a case of acute pancreatitis in the first trimester. A 36-year-old primigravida at 11 weeks of gestation complained of severe lower abdominal pain. The pain gradually worsened and migrated toward the epigastric region. She had no history of chronic alcoholism. Blood investigations showed elevated level of C-reactive protein (9.58 mg/dL), pancreatic amylase (170 IU/L), and lipase (332 IU/L). There was no gallstone and no abnormality in the pancreatic and biliary ducts on ultrasonography. Antinuclear antibody and IgG4 were negative and no evidence of hyperlipidemia or diabetes was found. There was also no evidence of viral infection. On the third day of hospitalization, she was diagnosed with severe acute pancreatitis on magnetic resonance imaging. Medical interventions were initiated with nafamostat mesilate and ulinastatin, and parenteral nutrition was administered through a central venous catheter. On the eighth day of hospitalization, her condition gradually improved with a decreased level of pancreatic amylase and the pain subsided. After conservative management, she did not have any recurrence during her pregnancy.

## 1. Introduction

The incidence of acute pancreatitis in pregnancy has been reported to be approximately 1 in 1000 to 1 in 10,000 pregnancies [1]. Previously, acute pancreatitis during pregnancy was a serious condition and the maternal mortality rate was high, but the mortality rate has recently decreased because diagnosis is reached earlier and maternal and neonatal intensive care have improved. However, studies have shown that cases of maternal death and fetal demise still occur [2, 3]. More than 50% of cases during pregnancy are diagnosed in the third trimester, and acute pancreatitis is more common with advancing gestational age [4–6]. Here, we report a case of acute pancreatitis in the first trimester of pregnancy. The patient recovered and delivered a healthy baby at term.

## 2. Case Report

The patient was a 36-year-old woman who was gravida 0 para 0. She had laparoscopic surgery 3 years ago for a ruptured endometriotic ovarian cyst at our hospital. She underwent intrauterine insemination and became pregnant. From 6 weeks of gestation, she was diagnosed with hyperemesis and was treated with herbal medicines prescribed by her physician and fluid infusions. When she was at 11 weeks of gestation, she was admitted to the hospital because of abdominal pain and vomiting that had begun after dinner, several hours before admission. She had neither vaginal bleeding nor diarrhea.

On admission, she was conscious with a body temperature of 37.0°C, pulse of 84 beats/min, and a blood pressure of 108/61 mmHg. Ultrasound examination showed an intrauterine gestational sac with a fetus, and the fetal heart beat was regular. There was a 20.0 × 9.0 mm hypoechoic lesion that was presumed to be subchorionic hemorrhage. There was neither ovarian tumor nor intra-abdominal fluid collections. She was provisionally diagnosed with a threatened miscarriage and subchorionic hemorrhage and was given isoxsuprine hydrochloride intravenously. However, the epigastric pain gradually worsened and was accompanied by an increase in body temperature to 38.9°C within several hours. To control the pain, she was given 15 mg of pentazocine hydrochloride by an intramuscular injection. The possibility of appendicitis was excluded because there was no tenderness at McBurney's point.

On the day of admission, her white blood cell count (WBC) was 7480/*μ*L and C-reactive protein (CRP) was 0.92 mg/dL. The findings of blood analysis that was performed on the next morning of hospitalization are shown in Table 1. WBC was still within the normal range (7020/*μ*L) but CRP was raised to 9.58 mg/dL. Her serum amylase was 201 U/L and pancreatic amylase and lipase were increased to 170 U/L and 332 IU/L, respectively. No evidence of hyperlipidemia and diabetes was found. We suspected acute pancreatitis from her laboratory data, but ultrasonography showed no typical findings of acute pancreatitis such as pancreatic enlargement or inflammatory changes around the pancreas. Antinuclear antibody and IgG4 were negative. In addition, antibodies against viruses such as hepatitis B and C viruses, cytomegalovirus, respiratory syncytial virus, adenovirus, mumps virus, coxsackie viruses B1 to B6, and Epstein-Barr virus were all negative (Table 1). On the second day of hospitalization, her epigastric pain persisted and CRP had increased to 15.5 mg/dL, so she underwent magnetic resonance imaging (MRI) examination.

**Figure 1:**
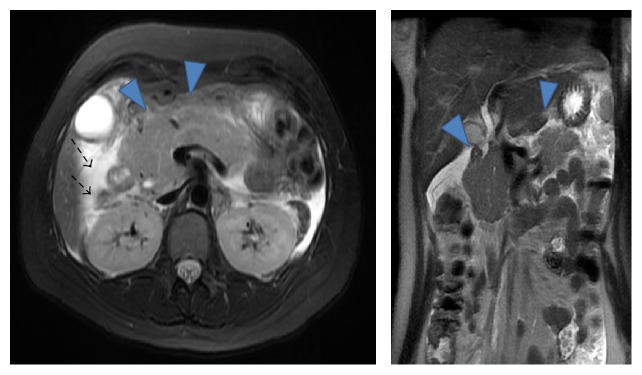
MRI T2W. (▼) Enlargement of the pancreatic body. (→) Inflammation of acute pancreatitis, extending to fat tissue and reaching beyond the lower pole of the kidney.

MRI showed enlargement of the pancreatic body and the inflammation extended to the fat tissue, reaching beyond the lower pole of the kidney (Figure 1). Because computed tomography or a contrast study is not recommended for pregnant women, especially in the first trimester, the diagnosis of acute pancreatitis was made based on the findings of MRI.

**Figure 2:**
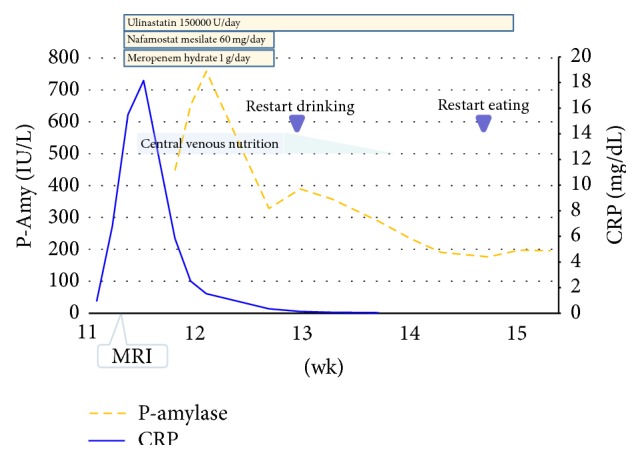
Clinography after hospitalization. Serum levels of CRP and pancreatic amylase (P-Amy) are shown. Medical interventions are shown in the graph.

Because there were no bile-pancreatic duct or lower bile duct stones, biliary pancreatitis was ruled out. We carried out conventional therapy including nutritional support, fluid infusion, antibiotics (meropenem hydrate), and protease inhibitors (nafamostat mesilate and ulinastatin). Her epigastric pain gradually reduced after the initiation of medical intervention for acute pancreatitis and some parameters on blood examination improved after a few days. She completely recovered and was discharged at 19 weeks of gestation. Her serum amylase levels had dropped to 149 U/L at discharge. Her CRP levels, pancreatic amylase levels, and medical interventions during her hospitalization are summarized in Figure 2.

After conservative management, she did not have any recurrence of acute pancreatitis. At 22 weeks of gestation, she was diagnosed with threatened preterm labor triggered by per vaginal bleeding and was again hospitalized and underwent continuous intravenous infusion of ritodrine hydrochloride. At 37 weeks + 2 days of gestation, cesarean section was performed because of marginal placenta previa. The baby's birth weight was 2694 g and the physical examination and clinical findings were normal.

## 3. Discussion

Acute pancreatitis is a common disease in the general population with an annual incidence of 27.7 per 100,000. Gallstones and alcohol abuse are the factors most strongly associated with acute pancreatitis in Japan. Because men often drink more alcohol than women, alcoholic-related pancreatitis appears more often in men. On the other hand, gallstone pancreatitis occurs more often in women because of the higher incidence of gallstones in women. Acute pancreatitis in pregnancy occurs infrequently and has a reported incidence of approximately 1 in 1,000 to 1 in 10,000 pregnancies [1]. The incidence of acute pancreatitis increases with gestational age; 53% to 79% of these cases were diagnosed in the third trimester [3, 7]. The incidence of acute pancreatitis in the first trimester is lower than that in the third trimester and its incidence has been reported to be 19% [7] or 29% [8]. Tang et al. reported that, among pregnant women with pancreatitis, the percentage of women who reached term was the lowest for those who developed pancreatitis in the first trimester and these women were at highest risk of fetal loss and preterm delivery because the fetus in the first trimester is vulnerable [8]. In contrast, Legro and Laifer reported that the prognosis for pregnancy outcome in the first trimester was good [9]. Indeed, reaching the correct diagnosis of acute pancreatitis in the first trimester is more difficult than in the third trimester of pregnancy. The symptoms of acute pancreatitis such as vomiting and abdominal pain are similar to symptoms of hyperemesis or threatened abortion. When acute pancreatitis becomes worse and ascites is pooled in the pelvic cavity, it is difficult to distinguish it from ectopic pregnancy or ruptured corpus luteal cyst. We first assumed that abdominal pain was caused by uterine contraction and vomiting was caused by hyperemesis gravidarum; thus, we diagnosed her with threatened abortion. However, because abdominal pain was not improved after hospitalization, gastrointestinal disease was suspected and the measurement of amylase levels enabled us to reach the diagnosis of pancreatitis.

Compared with the nonpregnant general population, acute pancreatitis in pregnant women is more likely to be associated with cholelithiasis, while hyperlipidemia and alcohol abuse as causes are less frequent [10]. Previous studies showed that 65% or more cases of acute pancreatitis in pregnancy were associated with cholelithiasis [2, 4, 11].

Women are likely to have gallstones during pregnancy. Cholesterol secretion in the hepatic bile increases in the second and third trimester of pregnancy, and the increase in the percentage of cholic acid leads to the increase of supersaturated bile. Moreover, elevated levels of progesterone during pregnancy induce smooth muscle relaxation of the gallbladder, which causes bile stasis. Increased cholesterol concentration in the hepatic bile and decreased emptying of the gallbladder lead to the retention of cholesterol crystals, and gallstones eventually form. In addition, in the third trimester, an enlarged uterus and increased intra-abdominal pressure on the biliary duct increase the risk of acute pancreatitis [1, 8, 12]. On the other hand, pancreatitis induced by hyperlipidemia is also observed during pregnancy. In this situation, serum triglyceride increases above 1000 mg/dL because of the increased secretion of triglycerides, and production of very low-density lipoproteins is induced by elevated levels of estrogens. It is reported that hyperlipidemic pancreatitis in pregnancy occurs more frequently and is more severe in the setting of familial hyperlipidemia [13]. In our case, the patient was not a habitual alcohol drinker. Also, there was no definite evidence of intra- or extrahepatic duct dilatation, gallstones, or pancreaticobiliary maljunction.

Viral infection could be a cause of acute pancreatitis. We first suspected viral infection because antinuclear antibody and IgG4 were negative, and no evidence of hyperlipidemia or diabetes was found. However, virus antibodies such as hepatitis B or C virus, cytomegalovirus, respiratory syncytial virus, adenovirus, mumps virus, coxsackie viruses B1 to B6, and Epstein-Barr virus were all negative. The cause of her acute pancreatitis remains unclear; thus, we diagnosed her with idiopathic acute pancreatitis. Geng et al. reported that 11.1% of patients with acute pancreatitis during pregnancy had idiopathic disease [14].

Although the maternal and fetal mortality rates in acute pancreatitis are declining because of earlier diagnosis and advances in treatment options, fetal loss could still occur. Hernandez et al. reported that 4.7% of pregnant women lost their fetus, based on experience of a single center over a 10-year period [15]. The risks to the fetus during acute pancreatitis are not only in utero fetal death but also threatened preterm labor and prematurity. Termination of pregnancy could be an option to cure acute pancreatitis during pregnancy.

Acute pancreatitis in pregnancy is rare, but this disease could be the cause of acute abdominal pain and manifest with emesis-like symptoms during the first trimester. In our case, analysis of amylase levels and MRI helped us to reach the correct diagnosis. We should consider this disease when pregnant women complain of severe abdominal pain with symptoms related to the alimentary tract.

## Figures and Tables

**Table 1:** Laboratory data of blood analysis on the next morning of hospitalization and additional data.

Data on the next morning of hospitalization	

WBC	7020/*μ*L
RBC	4.22 × 10^6^/*μ*L
Hb	13.1 g/dL
Hct	36.70%
Plt	210 × 10^3^/*μ*L
Neutrophils	85.40%
Eosinophils	0.40%
Lymphocytes	7.90%
PT%	76.00%
PT-INR	1.16
APTT	33.6 sec
Fibrinogen	477 mg/dL
D-dimer	11.0 *μ*g/mL
TP	7.1 g/dL
Alb	4.3 g/dL
T-Bil	1.4 mg/dL
AST	14 IU/L
ALT	12 IU/L
BUN	5.7 mg/dL
Cr	0.37 mg/dL
CRP	9.58 mg/dL
PCT	0.36 ng/mL
Amylase	201 U/L
P-amylase	170 IU/L
Lipase	332 IU/L
LDH	180 U/L
Alp	166 U/L
*γ*-GTP	25 U/L
ChE	159 mg/dL
Cholesterol	136 mg/dL
Triglyceride	67 mg/dL
LDL cholesterol	70 mg/dL
HDL cholesterol	53 mg/dL
FBS	96 mg/dL

Additional data	

CA19-9	26 U/mL
CEA	1.2 ng/mL
ANA	>40-fold
IgG	1124 mg/dL
IgG4	58 mg/mL
IgM	55 mg/dL
IgA	243 mg/dL
HBs antigen	(−)
HCV antibody	(−)
HIV antibody	(−)
TPHA	(−)
HTLV-1	(−)
CMV-IgG	>4-fold
CMV-IgM	0.01 C.O.I
RS virus antibody	>4-fold
Anti-adenovirus antibody	4-fold
Mumps virus antibody IgG	(±)
Mumps virus antibody IgM	(−)
Anisakis larvae antibody	(−)
Coxsackie virus B1~B6	>4-fold
Anti-rubella antibody HI	256-fold
EBV-IgG	(−)

WBC: white blood cells; RBC: red blood cells; Hb: hemoglobin; Hct: hematocrit; Plt: platelet; PT: prothrombin time; INR: international normalized ratio; APTT: activated partial thromboplastin time; TP: total protein; Alb: albumin; T-Bil: total bilirubin; AST: aspartate transaminase; ALT: alanine aminotransferase; BUN: blood urea nitrogen; Cr: creatinine; CRP: C-reactive protein; PCT: procalcitonin; LDH: lactate dehydrogenase; Alp: alkaline phosphatase; *γ*-GTP: *γ*-glutamyl transpeptidase; ChE: cholinesterase; FBS: fasting blood sugar; CA19-9: carbohydrate antigen 19-9; CEA: carcinoembryonic antigen; ANA: antinuclear antibody; TPHA: *Treponemapallidum* hemagglutination test; CMV: cytomegalovirus; C.O.I: cutoff index; HI: hemagglutination inhibition test; EBV: Epstein-Barr virus.
